# Bioorthogonal Reactions in Activity-Based Protein Profiling

**DOI:** 10.3390/molecules25245994

**Published:** 2020-12-18

**Authors:** Steven H. L. Verhelst, Kimberly M. Bonger, Lianne I. Willems

**Affiliations:** 1 Laboratory of Chemical Biology, Department of Cellular and Molecular Medicine, KU Leuven, Herestr. 49, Box 802, 3000 Leuven, Belgium; 2 AG Chemical Proteomics, Leibniz Institute for Analytical Sciences ISAS, e.V., Otto-Hahn-Str. 6b, 44227 Dortmund, Germany; 3 Institute for Molecules and Materials, Radboud University, Heyendaalseweg 135, 6525 AJ Nijmegen, The Netherlands; 4 York Structural Biology Laboratory, Department of Chemistry, University of York, York YO10 5DD, UK

**Keywords:** activity-based probes, activity-based protein profiling, bioorthogonal chemistry, chemoselective ligation, click chemistry, covalent inhibitors, enzyme probes, target identification

## Abstract

Activity-based protein profiling (ABPP) is a powerful technique to label and detect active enzyme species within cell lysates, cells, or whole animals. In the last two decades, a wide variety of applications and experimental read-out techniques have been pursued in order to increase our understanding of physiological and pathological processes, to identify novel drug targets, to evaluate selectivity of drugs, and to image probe targets in cells. Bioorthogonal chemistry has substantially contributed to the field of ABPP, as it allows the introduction of tags, which may be bulky or have unfavorable physicochemical properties, at a late stage in the experiment. In this review, we give an overview of the bioorthogonal reactions that have been implemented in ABPP, provide examples of applications of bioorthogonal chemistry in ABPP, and share some thoughts on future directions.

## 1. Introduction

Approximately two decades ago, several seminal papers from the groups of Cravatt and Bogyo started the field of activity-based protein profiling (ABPP) [1,2,3,4]. ABPP was initially reported as a technique to detect active enzymes in a complex proteome, such as a cell lysate or a whole cell, with in-gel or immunoblot detection after sodium dodecyl sulfate polyacrylamide gel electrophoresis (SDS-PAGE). In the past two decades, the application areas have vastly expanded, accelerated by the implementation of bioorthogonal chemistry. This review is dedicated to discussing the topic of bioorthogonal chemistry in ABPP and highlighting recent advances in this field.

**Figure 1:**
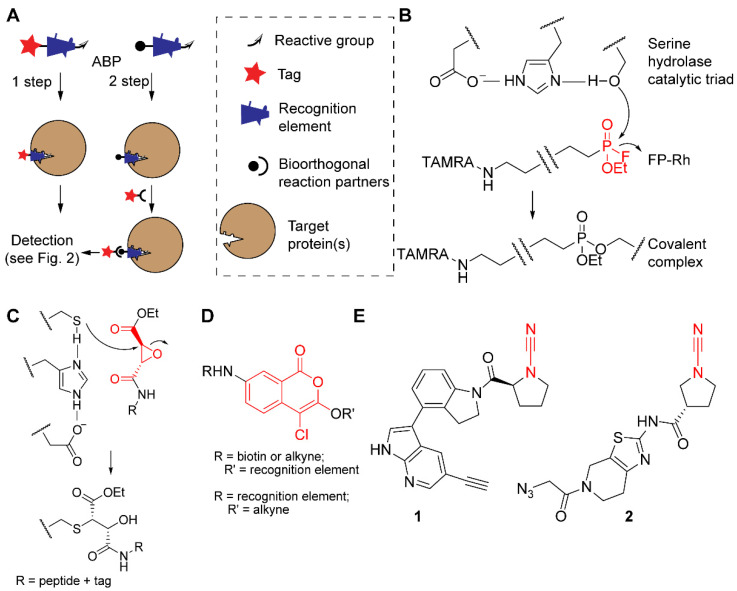
The concept of ABPP and examples of probes and reactive groups. (**A**) Schematic representation of one- and two-step ABPs with their three structural elements (tag or bioorthogonal handle, recognition element, and reactive group) and binding of a target protein by the recognition element. (**B**) Example of the fluorophosphonate probe FP-Rh (reactive group in red) and the covalent reaction mechanism with the active site of a serine hydrolase. (**C**) Example of epoxysuccinate probes (reactive group in red) and the covalent reaction mechanism with the active site of a cysteine protease. (**D**) Structure of probes based on the 4-chloro-isocoumarin scaffold (in red) for serine proteases, with tags and recognition elements at different positions. (**E**) Two examples of cyanimide probes for the DUB UCHL1 (reactive cyanimide group in red).

ABPP makes use of chemical probes that covalently bind to an active target enzyme or enzyme family but not to inactive counterparts. They do so by making use of a mechanism-based reaction with active site residues. These activity-based probes (ABPs) (see Figure 1A) usually consist of three elements.

**Figure 2:**
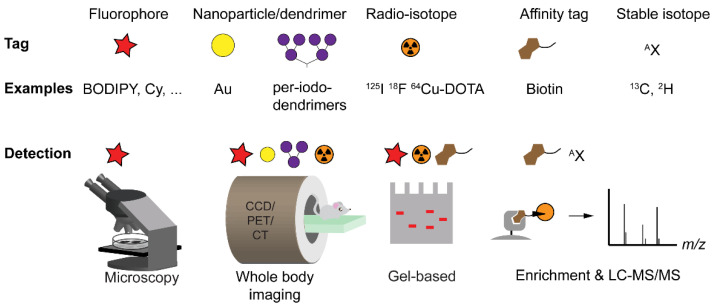
Detection strategies in ABPP. Overview of different types of tags with examples and techniques for detection. CCD: charge-coupled device camera (optical), PET: positron emission tomography, CT: X-ray computed tomography, LC-MS/MS: liquid chromatography with tandem mass spectrometry. Note that fluorescence detection in whole body imaging can only detect signal generated several millimeters under the skin due to the limited tissue penetration of light.

(1) A reactive group that forms a covalent bond with the target protein. In the case of enzymes that use a nucleophilic amino acid side chain for attack onto their substrates, the reactive group is usually an electrophile. For example, most serine hydrolases (SHs), a superfamily of enzymes that catalyze the breakdown of amide and ester bonds [5], react with the fluorophosphonate electrophile present in the ABP FP-Rh (Figure 1B) [6,7]. Because the nucleophilic attack onto the electrophilic trap resembles the first step of the catalytic mechanism, these probes are considered to be truly ‘activity-based’. Consequently, inactive enzyme states, such as zymogen or inhibitor-bound forms, will not react with the ABPs. The reactive group can lead to some degree of probe selectivity, e.g., fluorophosphonates (Figure 1B) only react with activated serine residues [1], whereas epoxysuccinates (Figure 1C) selectively react with cysteine proteases [3]. If the target protein does not contain a nucleophilic residue in its active site, a photoaffinity labeling (PAL) strategy can be used. Here, covalent bond formation is ensured by a photoreactive group, such as a diazirine or a benzophenone. These probes are generally referred to as affinity-based probes (AfBPs). We will not discuss AfBPs in detail and refer for the background of PAL and its applications to a review that appeared elsewhere [8].
(2) A spacer or recognition element that induces a higher degree of selectivity to the target protein. Together with the reactive group, it will determine which target proteins are covalently modified [9]. Often, recognition elements are derivatives of the substrate, such as short stretches of amino acids (for proteases), modified mono- or polysaccharides (for glycosidases), or even whole proteins (for deubiquitinating enzymes; DUBs). The recognition element may also originate from a known inhibitor. For example, 4-chloro-isocoumarin (Figure 1D), a common heterocyclic scaffold used for covalent inhibition of serine proteases [10], has been equipped with biotin, alkyne, or fluorophores to target various soluble serine proteases [11,12], the intramembrane protease family of rhomboids [13,14], as well as acyl protein thioesterase, a serine hydrolase that cleaves fatty acids from S-acylated cysteine residues [15]. Other examples include cyanimides **1** [16] and **2** [17], derived from inhibitors of ubiquitin carboxy-terminal hydrolase L1 (UCHL1), a DUB associated with various human diseases, including neurodegeneration and cancer. Interestingly, these small-molecule ABPs are cell permeable, allowing the study of DUBs in a cellular environment [16,17] and even in an in vivo zebrafish embryo animal model [17].
(3) A tag that enables detection of the covalent probe-protein complex [18]. Various tags have been used in ABPP, including biotin, fluorophores, radioisotopes, stable isotopes, and nanoparticles. The introduced tag determines which possibilities are available for read-out. Many studies use the abovementioned tags for visualization of biomolecules after separation by SDS-PAGE. Other possible detection methods include fluorescence microscopy, tandem mass spectrometry (after biotin-mediated enrichment), positron emission tomography (PET), and X-ray computed tomography (CT) (see Figure 2).

Placing a tag onto an ABP can have a pronounced effect on the physicochemical properties and biological behavior of the probe. Notorious is the effect of attaching biotin, which generally renders a probe cell impermeable. Fluorophores with charged functional groups can have the same effect. In addition, some tags are quite bulky and may cause a steric clash, preventing the probe from effectively entering the enzyme active site. The installment of small “mini-tags”, such as an azide or an alkyne, can prevent the abovementioned shortcomings. By making use of bioorthogonal chemistry, these mini-tags can react in a second step with a molecule carrying a suitable tag for detection.

In this review, we will describe the use of bioorthogonal reactions within the field of ABPP. In Section 2, we first give an overview of the different bioorthogonal ligation reactions and illustrate this discussion with specific examples of their use in ABPP. In Section 3, we discuss which mutually orthogonal combinations of bioorthogonal reactions have been made within ABPP to enable the simultaneous labeling of multiple enzyme activities. Section 4 is dedicated to specific applications of bioorthogonal reactions in ABPP. Finally, we reflect on future directions in Section 5.

## 2. Overview of Bioorthogonal Reactions in ABPP

The field of bioorthogonal chemistry uses chemical transformations that can selectively take place in complex biological samples without reacting or interfering with any of the native biological components or processes. As such, these reactions need to be chemoselective, use reactants that are abiotic and inert towards the many other functionalities in biomolecules, and should ideally produce no (toxic) by-products. In addition, bioorthogonal reactions should proceed readily in mild aqueous conditions, at physiological pH, and at low reagent concentrations. Despite these challenges, the field has made great progress since it was first pioneered in the late 1990s [19,20]. In these early reports, ketones were introduced into cell surface glycans [19] or proteins [20] and subsequently labeled with fluorescent or biotin-tagged hydrazide reagents. Although these reactions do not fulfill all criteria of bioorthogonality (i.e., ketones are not abiotic), they represented an important first step into the development of biocompatible chemical transformations.

**Figure 3:**
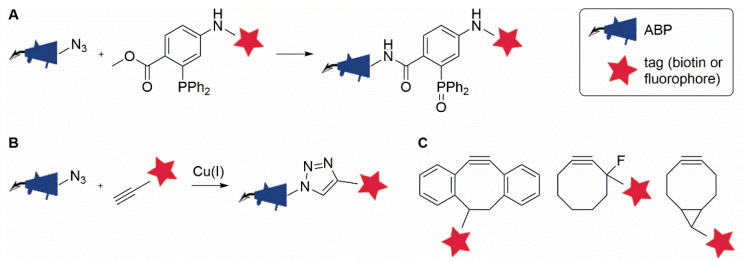
Azide-based bioorthogonal ligation reactions that have been used in combination with ABPP. (**A**) Staudinger–Bertozzi ligation between an azide and a phosphine. (**B**) Copper(I)-catalyzed azide-alkyne cycloaddition (CuAAC, or ‘click’ reaction). (**C**) Strained alkynes for use in copper-free strain-promoted azide-alkyne cycloaddition (SPAAC).

A few years later, Bertozzi and co-workers improved upon their previous approach by designing a carbohydrate derivative equipped with an azide as a mini-tag. After metabolic incorporation into cell surface glycans, the azide can undergo selective ligation with a biotin-tagged phosphine reagent, a bioorthogonal reaction that has become known as the Staudinger–Bertozzi ligation [21] (Figure 3A). Over the following two decades, an expanding repertoire of different reagents and transformations havw been explored, with over 20 types of bioorthogonal ligation reactions reported to date (see for recent reviews [22,23]). These reactions have been used to label various biomolecules, making use of suitably tagged metabolic precursors to introduce desired mini-tags into glycans [24], proteins [25], lipids [26], and nucleic acids [27]. In addition, bioorthogonal chemistry has also found application in other contexts, including drug delivery [28], radiolabeling [29], and in the field of ABPP [30,31,32]. The various types of bioorthogonal ligations that have been used in the context of ABPP will be described below.

### 2.1. The Staudinger–Bertozzi Ligation

In 2003, two studies by different groups revealed for the first time that bioorthogonal chemistry can be implemented into ABPP strategies [33,34]. Both groups used APBs equipped with an azide mini-tag while they applied different ligation reactions to label the azide-tagged probe-enzyme conjugates. Speers et al. used a copper(I)-catalyzed azide-alkyne cycloaddition (CuAAC) to label enzyme activity in cell extracts (Figure 3B) [33]. This reaction will be further discussed in Section 2.2. In contrast, Ovaa et al. used the Staudinger–Bertozzi ligation to label proteasome activity in cell extracts (Figure 3A) [34]. The two-step labeling strategy permitted the targeting of active proteasome subunits in living cells using a vinyl sulfone-based ABP, and subsequent labeling of the probe-bound enzymes in cell extracts with biotin, a tag that would otherwise have made the ABP poorly cell permeable. A different azide-tagged proteasome probe, carrying an epoxyketone as the reactive group rather than a vinyl sulfone, was later used to relatively quantify the activities of the various proteasome subunits in cells by making use of this two-step labeling approach [35].

The Staudinger–Bertozzi ligation has also been applied to label several other enzyme classes, including cysteine proteases [36] and retaining glycoside hydrolases [37,38]. Vocadlo and Bertozzi [37] and Stubbs et al. [38] designed ABPs based on known fluorosugar inhibitors for retaining β-glycosidases and *N*-acetylglucosaminidases, respectively. In both cases, the authors argued that the introduction of a bulky biotin reporter group directly onto the carbohydrate structure would likely interfere with binding of the resulting probes in the active sites of their target enzymes. In contrast, azide tags were accommodated well by the tested enzymes. The resulting probes represented the first examples of ABPs that enable the labeling of glycosidase activity in cell extracts. Interestingly, a comparative study by Witte et al. [39] later revealed that certain glycosidases appear to be very tolerant towards larger modifications, in particular the addition of BODIPY fluorophores, onto similar inhibitor scaffolds.

The main drawback of the Staudinger–Bertozzi ligation is its relatively slow reaction kinetics [40,41], a limitation that has prompted researchers to pursue new faster ligation reactions. Yet, Verdoes et al. were able to show, using an elegantly designed ABP carrying both a fluorescent and an azide tag, that the Staudinger–Bertozzi ligation can proceed to completion under the right reaction conditions, even within the steric constraints of an enzyme active site [42]. A number of studies have made direct comparisons between the Staudinger–Bertozzi ligation and azide-alkyne cycloadditions as well as inverse electron demand Diels–Alder reactions (see Section 2.2 and Section 2.3) within the context of ABPP in cell extracts [43,44]. Both studies found that the Staudinger–Bertozzi ligation was indeed slower, requiring much higher concentrations of the phosphine reagent than an alkyne or tetrazine, but it was also the most selective. Perhaps largely due to its excellent selectivity, this reaction has been widely used for bioorthogonal labeling, especially in cells and in vivo [41].

### 2.2. The Azide-Alkyne Cycloaddition

The small size of the azide tag, its inertness towards biological functionalities, and the excellent stability during chemical probe synthesis make it an ideal mini-tag and a popular choice for a variety of applications [45]. The azide displays unique reactivity: In addition to its ability to react in Staudinger ligations, it can also undergo a 1,3-dipolar cycloaddition reaction with an alkyne, an equally small and abiotic functional group, giving a triazole product (Figure 3B). Whereas the uncatalyzed azide-alkyne cycloaddition requires high temperatures to proceed, seminal work by Sharpless and co-workers [46] and Meldal and co-workers [47] in 2002 demonstrated that great rate enhancements are achieved by using copper(I) as a catalyst. The reaction can now proceed at ambient temperatures and with excellent kinetics [48].

The first implementation of CuAAC in ABPP followed quickly. Speers et al. modified a phenyl sulfonate probe with an azide tag to enable the labeling of enzyme activities in extracts from living cells treated with the azide-tagged ABP and even in tissue homogenates from probe-treated mice [33]. Since then, CuAAC has been used in myriads of other ABPP studies, covering a wide range of enzyme classes including serine hydrolases, cysteine proteases, DUBs, arginine deiminases, kinases, cytochrome P450 enzymes, and glycosidases (see, for a recent review, [31]).

Interestingly, the CuAAC reactants can be easily interchanged as both the azide and the alkyne are small and therefore not likely to perturb the binding properties of the probe. As such, the azide can be part of the ABP and combined with an alkyne-conjugated tag as reaction partner, or it can be part of the reporter group when a probe with an alkyne functionality is used. Although both options are regularly applied, several studies have shown that an azide-tagged fluorophore gives slightly lower background labeling than an alkyne-conjugated fluorophore during the CuAAC reaction [43,49].

CuAAC is arguably the most frequently used bioorthogonal labeling reaction across a range of applications. It is particularly useful for in vitro applications, for example in inhibitor discovery or target identification (see Section 4). Over the past decade, efforts have been made to reduce the cellular toxicity of the copper-catalyzed ligation by using copper-stabilizing ligands with increased water solubility [50,51], ligands that form less toxic copper complexes [52], as well as copper-chelating azides [53,54,55]. These approaches work by accelerating the reaction, thereby reducing the required concentration of the copper catalyst, and/or preventing the formation of reactive oxygen species so that labeling reactions can occur inside living cells or even in vivo.

In 2004, an important improvement of the azide-alkyne cycloaddition reaction was reported. It was shown that cyclooctynes (Figure 3C) readily react with azides in the absence of a metal catalyst due to the relief of ring strain that drives the reaction [56,57,58,59]. These strain-promoted azide-alkyne cycloadditions (SPAACs) have not been extensively used in the context of ABPP. Cyclooctynes are known to be reactive towards free thiols [60,61,62], which precludes their use in complex samples, such as lysates or the intracellular environment. Indeed, it was found that these reagents cause large amounts of nonspecific background labeling when they are reacted with azide-tagged ABPs in cell extracts [43,44,63]. This non-specific reactivity, however, can be partially resolved by masking cysteine residues using, for example, iodoacetamide [63]. Nonspecific labeling by cyclooctynes was not observed in an ABPP study with probes targeting a gastric proton pump [64]. In this case, labeling was performed in membrane fractions, which likely have a lower abundance of free thiols than whole cell extracts. Thus, in such cases, SPAAC may well be a useful approach for two-step ABPP, although the authors reported superior labeling efficiency when using tetrazine-based bioorthogonal chemistry (see Section 2.3) compared with SPAAC.

### 2.3. The Inverse Electron-Demand Diels-Alder Ligation

**Figure 4:**
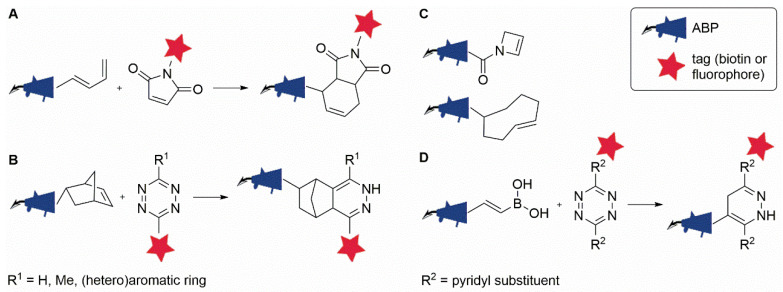
Diels–Alder-type bioorthogonal ligation reactions that have been used in combination with ABPP. (**A**) Diels–Alder ligation. (**B**) Inverse electron-demand Diels–Alder ligation (iEDDA) between a probe equipped with norbornene and a tetrazine-conjugated (fluorescent/biotin) tag. (**C**) Acylazetine and *trans*-cyclooctene mini-tags for iEDDA with tetrazine reagents. (**D**) Selective iEDDA between vinylboronic acid-modified probes and dipyridyl-substituted tetrazines.

The Diels–Alder (DA) reaction is a [4 + 2] cycloaddition between a conjugated diene and a dienophile that is widely used in synthetic organic chemistry for the construction of six-membered (hetero)cycles. To achieve optimal reaction rates, an electron-poor dienophile, such as maleimide, should be reacted with an electron-rich diene—a reaction referred to as the ‘normal electron-demand’ DA reaction (Figure 4A). The fact that the DA reaction displays a rate acceleration in water [65,66] and often proceeds readily in the absence of a catalyst has stimulated interested in its use as a bioorthogonal ligation reaction.

An initial application of the DA reaction in two-step ABPP involved the reaction between a maleimide-conjugated fluorophore and several diene-modified ABPs for the labeling of proteasome activity [67] (Figure 4A). A major drawback of this approach is the inherent reactivity of maleimide towards thiols. In fact, maleimides are common bioconjugation tools used for the modification of cysteine residues in proteins [68]. Even though background labeling by the maleimide reagent was substantially reduced upon the masking of cysteine residues [67], this side reaction makes the approach less useful as a bioorthogonal ligation reaction.

A solution to this problem was presented by the advance of the ‘inverse electron-demand’ DA reaction (iEDDA) between an electron-rich dienophile and an electron-poor diene (Figure 4B), as pioneered by two independent groups in 2008. Blackman et al. reported the use of the cycloaddition between an electron-poor diene, tetrazine, and *trans*-cyclooctene (TCO) as the dienophile for protein bioconjugation [69], while Devaraj et al. used fluorescently tagged tetrazines to image norbornene-tagged antibodies targeted to cell surface receptors on live cells [70]. The approach was quickly adapted to enable the intracellular imaging of microtubule-binding small molecules [71] and the labeling of tumor-targeting antibodies in vivo in a mouse model [72]. A few years later, this reaction was also introduced into the field of ABPP, enabling the two-step labeling of proteasome activity in living cells by using a norbornene-tagged ABP (Figure 4B) [43].

Thanks to its fast reaction kinetics, rivalling those of CuAAC, while retaining a superior level of selectivity, the iEDDA has attracted immense interest in the fields of bioconjugation and bioorthogonal chemistry [73]. A variety of dienophiles and a range of differently substituted tetrazines have been reported in the literature, as well as a number of ‘turn-on’ probes that make use of the convenient ability of tetrazines to quench conjugated fluorophores and thereby achieve fluorogenicity upon iEDDA reaction (see, for recent reviews, [74,75]). For the purpose of ABPP, a few different dienophiles have been described in addition to norbornene [43], including acylazetine [76], *trans*-cyclooctene [77], and vinylboronic acid [78] (Figure 4C,D). An interesting finding was made by Bonger and co-workers in their work on the use of vinylboronic acid as a dienophile in two-step ABPP (Figure 4D) [78,79]. Testing its reaction with a variety of different tetrazines, the authors found that the boronic-acid-conjugated alkene showed high levels of selectivity for dipyridyl-s-tetrazines over those carrying methyl or phenyl substituents, opening up the possibility to perform mutually orthogonal tetrazine ligations with vinylboronic acid and other less-selective dienophiles [78]. The combination of bioorthogonal ligation reactions for simultaneous labeling of multiple enzymes will be further explored in Section 3.

A few studies have performed direct comparisons between iEDDA and other bioorthogonal ligation reactions for two-step ABPP. Comparing the efficiency of tetrazine-based iEDDA with that of SPAAC using a set of structurally similar benzimidazole probes, Paresi et al. found that iEDDA showed superior results [64]. While Liu et al. reported reduced selectivity of the tetrazine ligation as compared to CuAAC when using ibrutinib derivatives for labeling of Bruton’s tyrosine kinase [80], Willems et al. found the opposite when targeting proteasome activity [43]. Comparing various β-lactone-based ABPs for the labeling of penicillin-binding proteins, Brown et al. concluded that the main factors influencing the reaction efficiency appear to be related to the accessibility of the tags to their bioorthogonal reaction partners, rather than the speed of the reaction itself [81]. The authors also emphasized the usefulness of both CuAAC and iEDDA in different types of applications.

## 3. Orthogonal Bioorthogonal Reactions in ABPP

Profiling the activity of multiple proteins simultaneously may reveal a more detailed understanding of cellular protein dynamics and working mechanisms than the study of single proteins. Using ABPs with appropriately chosen mini-tags, a two-step labeling approach benefitting from compatible bioorthogonal reactions allows the selective introduction of unique molecular functionalities on each protein-probe complex. Reaction orthogonality can be achieved by adopting a variety of design principles, including differences in reaction mechanism, sterics of the reactants, reaction kinetics, or making use of activatable reactants.

Willems et al. demonstrated a single-pot labeling approach of three individual proteasome subunits using ABPs that are equipped with an azide, a norbornene, or an alkyne mini-tag [43]. The azide and norbornene firstly react by Staudinger–Bertozzi ligation and iEDDA with reporters containing a phosphine and tetrazine ligation handle, respectively. A third orthogonal reaction was accomplished by sequential addition of an azide that reacts with the alkyne upon addition of a copper catalyst.

Alternatively, Eising et al. used proteasome ABPs equipped with a norbornene and a vinyl boronic acid [78]. Here, the vinyl boronic acid specifically reacts with tetrazines containing a boron-coordinating substituent, while norbornene reacts with all tetrazines regardless of the substituent type. Selective ligations with the vinyl boronic acid and norbornene were achieved by using tetrazine reporters containing a pyridyl and a pyrimidyl substituent, respectively.

**Figure 5:**
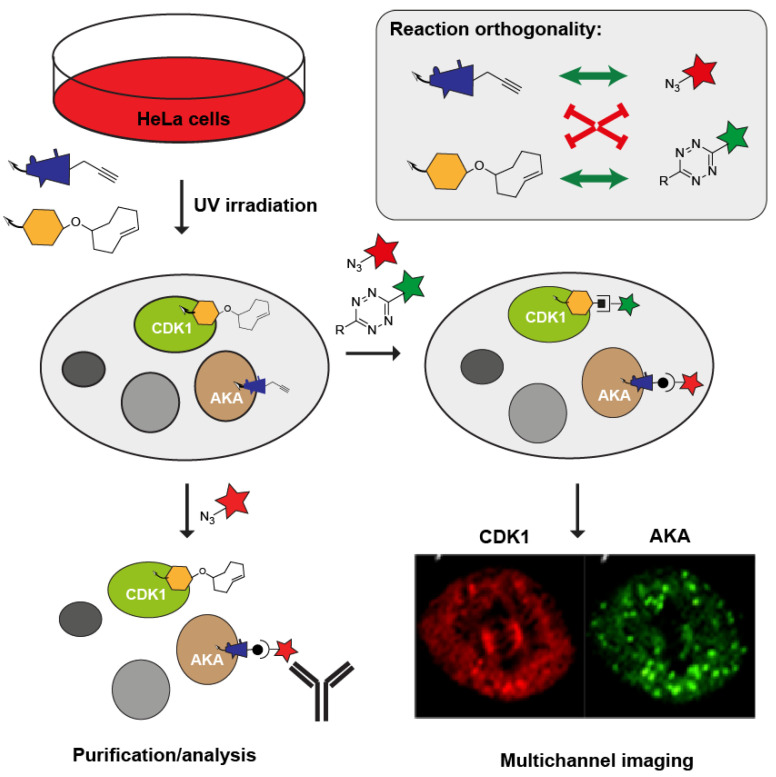
Orthogonality between bioorthogonal ligation reactions enables the simultaneous profiling of multiple enzyme targets. Figure exemplifies the experiment performed by Su et al. demonstrating subcellular localization of two cell cycle regulating kinases, cyclin-dependent kinase 1 (CDK1), and Aurora Kinase A (AKA). By using a two-step labeling approach, the same experimental samples could be used for enrichment as well as for imaging of the proteins. Fluorescent image taken from [82].

In a slightly different approach, Su et al. studied the localization of two kinases, cyclin-dependent kinase 1 (CDK1) and aurora kinase A (AKA), during mitosis using AfBPs equipped with an alkyne and a TCO mini-tag [82]. After a two-step labeling approach using two fluorophores conjugated to an azide and a tetrazine, the authors evidenced localization of the two kinases during all five stages of mitosis as visualized by confocal fluorescence microscopy (Figure 5).

Combining multiple bioorthogonal reactions in a single pot has been explored extensively beyond the field of ABPP. Recent developments in bioorthogonal reactions and design principles needed to create orthogonality have been reviewed elsewhere [22,23,83].

## 4. Specific Applications of Bioorthogonal Reactions in ABPP

Bioorthogonal reactions are used for various applications within ABPP but are not always essential. Nevertheless, ABPs with bioorthogonal handles have seen an increase in their occurrence as this allows flexibility in the choice of a detection tag at a late stage of probe development and in the selection of experimental read-out possibilities. In the paragraphs below, some of the most common applications of two-step ABPP are discussed.

### 4.1. Profiling in Disease

From an early stage, ABPP has been applied to profile active enzyme populations related to (human) disease. Fluorescently labeled ABPs were used, for example, to profile cysteine cathepsin proteases in different stages of pancreatic cancer in mouse models [84] and serine hydrolases in cancer cell lines with different invasive properties [85]. While these studies with fluorescent ABPs show that bioorthogonal chemistry is not essential for such studies, the use of mini-tags can have some benefits. We will illustrate this with some more recent studies below.

**Figure 6:**
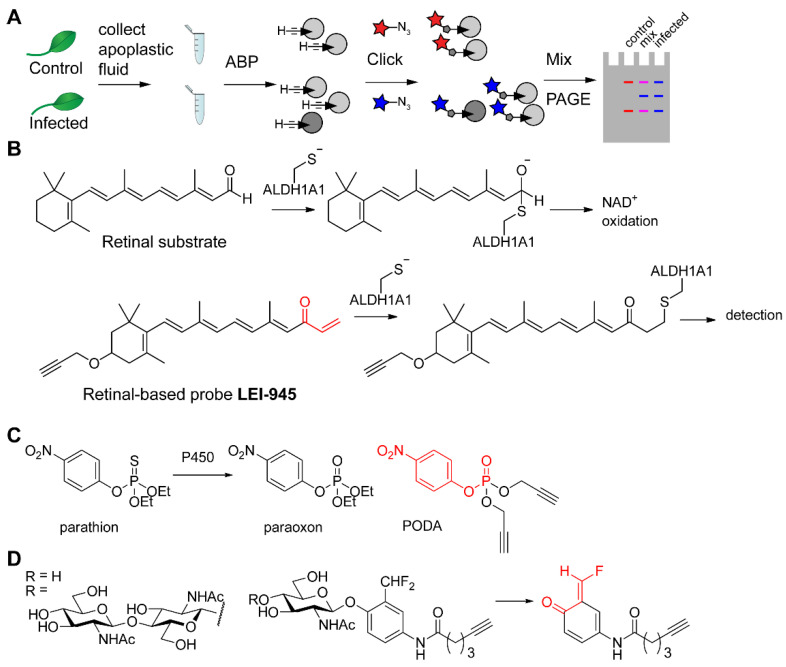
Profiling and discovery of enzymes by ABPP. (**A**) Schematic representation of DIGE-ABPP. Apoplastic fluid—or any other proteome—is labeled with a clickable probe, followed by CuAAC-mediated introduction of a different fluorophore in each sample. Next, samples can be mixed and analyzed. Whereas mixed colors (purple for both red and blue fluorophores) indicate similar activity of the probe targets, red or blue fluorescence reveals targets that are active only in control or infected sample. (**B**) Comparison of retinal with retinal-derived probe LEI-945 and its binding mechanism to aldehyde dehydrogenase ALDH1A1. (**C**) Structures of the pesticide parathion, the metabolite paraoxon, and the derived probe PODA. (**D**) *N*-Acetylglucosamine- and chitobiose-based ABPs and their binding mechanism: after cleavage of the carbohydrate moiety, a quinone-methide electrophile is formed, which reacts with a nearby nucleophile.

Hong and Van der Hoorn used differential gel electrophoresis ABPP (DIGE-ABPP) to compare the levels of active serine hydrolases in the apoplasts (intercellular spaces) of normal plant leaves versus those infected with *Pseudomonas syringae* [86]. Collection of apoplastic fluid from both leaves was followed by introduction of fluorophores of different colors by CuAAC, after which samples were mixed and separated by SDS-PAGE. The ratio of colors now reveals the difference in activity upon infection (Figure 6A). Although bioorthogonal ABPs are not necessary to reach the apoplast, it enables the use of the exact same probe in uninfected and infected leaves, thereby eliminating potential effects of the fluorophore on the physicochemical and biochemical properties of the probe.

The use of an alkyne handle in the design of an ABP for retinaldehyde dehydrogenases allowed Van der Stelt and co-workers to minimize the structural perturbation of the natural substrate retinal (Figure 6B). While the active site cysteine residue is trapped by the vinyl ketone electrophile, the propargyl ether allows subsequent introduction of a detection tag by CuAAC. As this probe is cell permeable, it enables profiling of aldehyde dehydrogenases in live cells. Specifically, the probe was used to compare different breast cancer cell lines after CuAAC and biotin/streptavidin-mediated enrichment followed by proteomics with label-free quantification [87].

### 4.2. Enzyme Discovery

Another application of ABPP is to functionally characterize enzymes for which knowledge of natural or artificial substrates is lacking. Reactivity towards an ABP will confirm that these enzymes have a functional active site with an intact catalytic machinery, and suggests that enzymes of unknown function must exhibit a certain catalytic activity in line with the specificity of the probe. Although these studies can be performed with classical probes, such as FP-Rh [7,88], probes with bioorthogonal handles have the advantage that they are more likely to be active in cellular environments as they more easily cross the cell membrane (in animals or derived cell lines) or the cell wall (in prokaryotes, fungi, and plants).

An interesting example of enzyme discovery has recently been reported for the extremophile *Sulfolobus acidocaldarius*, a species that was isolated from hot springs in the Yellowstone National Park (USA), and *Saccharolobus solfataricus*, found in the Solfatara vulcano (Italy). These species have optimal growth conditions at pH 2–3 and 75–80 °C, which may be detrimental to certain fluorescent tags. In vivo labeling with serine reactive fluorophosphonate and nitrophenyl phosphonate ABPs with alkyne tags led to the identification of several esterases, whose enzymatic activity was further confirmed in biochemical assays [89].

Lin et al. synthesized alkyne analogs of various organophosphate pesticides in order to discover their target proteins. Although acute toxicity of organophosphates is caused by inhibition of acetylcholine esterase, the complete range of proteins and affected biochemical pathways in exposed individuals is still poorly characterized. The use of small alkynes allows the ABP to be as close as possible to the original pesticide structure. For example, the ABP PODA only contains two more carbon atoms in comparison with paraoxon (Figure 6C), the species that is formed from parathion upon oxidation by cytochrome P450. The authors found a range of different serine hydrolases in mouse brain and mouse liver lysates that were targeted by the ABP—not only cholinergic targets, but also various lipases, peptidases, and carboxyesterases, depending on the profiled tissue [90].

In order to discover enzymes involved in microbial catabolism of chitin, Zegeye et al. used quinone-methide-forming probes based on *N*-acetylglucosamine and chitotriose (Figure 6D) [91]. Although these probes were used for labeling of bacterial secretomes, the alkyne handle on the probes allowed the flexibility to incorporate a fluorophore tag for convenient in-gel profiling of enzyme activities and a biotin tag for enrichment and identification of these enzymes. When cultures of the soil bacterium *Cellvibrio japonicus* were grown with different types of carbon sources (glucose, *N*-acetylglucosamine, or chitin), the secretome contained different chitin-catabolizing enzymes. Whereas absent in the glucose-containing conditions, the activity of five key chitinases was induced when grown on the chitin-containing medium.

### 4.3. Identification of Drug Targets and Off-Targets

**Figure 7:**
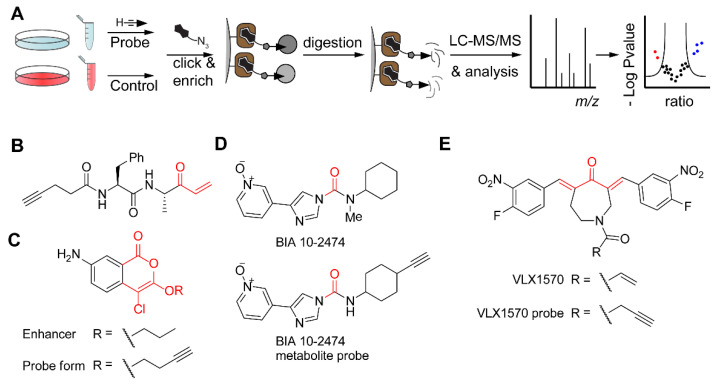
Target identification in ABPP by making use of bioorthogonal chemistry. (**A**) Schematic overview of a general workflow for target identification of ABPs. Live cells or a cell lysate are treated with a clickable probe. Recommended is a control experiment without a probe or with a competitive inhibitor. After cell lysis, CuAAC with a biotin-tagged reagent, and enrichment on immobilized streptavidin (in brown), tryptic digestion yields peptides for LC-MS/MS analysis and data processing, for example by volcano plot, as depicted. Note 1: the control experiment can be isotopically labeled, e.g., by SILAC (stable isotope labeling by amino acids in cell culture) or tryptic peptides can be labeled with heavy and light isotope labels before mixing. Alternatively, label-free quantification can be used. Note 2: Proteins may also be eluted prior to digestion in order to identify the modified peptide. Alternatively, after ‘on bead’ digestion, the modified peptide may be released by making use of, for example, a cleavable linker. (**B**) Alkyne-tagged ABP based on an inhibitor of attachment and invasion of the Toxoplasma parasite. The reactive electrophile is shown in red. (**C**) Structure of an enhancer of Toxoplasma invasion into host cells and an alkynylated derivative with the reactive electrophile in red. (**D**) Structure of FAAH inhibitor BIA 10-2474 and a probe derived from a de-methylated metabolite (electrophilic carbonyl in red). (**E**) Structure of DUB inhibitor VLX1570 and an alkyne-tagged probe form. Note that the two Michael acceptor systems (in red) can lead to crosslinking of proteins.

ABPP workflows that encompass enrichment and tandem MS-based proteomics have been implemented to identify targets and off-targets of drugs or drug candidates (Figure 7A). This requires covalent binding of the drug to its protein target, which can happen either by installing an electrophilic trap or by photo-affinity labeling. Overall, these studies can be divided into two strategies: (1) identification of the targets of inhibitors discovered in phenotypic screening, and (2) identification of off-targets of drugs that already have a known target. Additionally, the exact binding site of a drug may be identified, which provides information on the enzyme’s binding pocket or mechanism of action.

In order to shed more light on the process of invasion of the *Toxoplasma gondii* parasite into human host cells, the Bogyo laboratory performed phenotypic screening in the presence of a variety of cysteine and serine reactive compounds [92]. Several dipeptide analogs with Michael acceptors were found to block attachment to and invasion of host cells. An alkyne-tagged version of one of these compounds (Figure 7B) identified the protein TgDJ-1 as the target. Covalent modification of this protein leads to inhibition of secretion of the parasite microneme organelles, essential for invasion. Interestingly, the same screen also identified compounds that enhanced invasion of the parasites, in particular a serine hydrolase reactive isocoumarin (Figure 7C). Through a combination of target enrichment using an alkyne probe in live parasites and competition experiments using a broad-spectrum serine hydrolase probe, palmitoyl-protein thioesterase-1 was identified as the protein responsible for the increase in parasite invasion [93]. Although an invasive phenotype would be undesired in a therapeutical setting, it revealed the importance of protein lipidation in the invasion process and may lead to new therapeutic strategies.

The importance of off-target identification is underlined by several studies on BIA 10-2474 (Figure 7D), a fatty acid amide hydrolase (FAAH) inhibitor. In 2016, a phase I clinical trial with this compound resulted in the death of one volunteer. Whereas competitive ABPP with a serine hydrolase-targeting fluorophosphonate probe resulted in the identification of the serine hydrolase carboxyesterase 2 (CES2) as an off target [94], a probe derived from a major BIA 10-2474 metabolite (Figure 7D) was used for a more complete proteome-wide analysis of off-targets. Interestingly, this probe reacted with the catalytic cysteine of aldehyde dehydrogenases, which may contribute to the toxicity of BIA 10-2474. In addition, it emphasizes the benefit of derivatizing drugs (or their metabolites) with mini-tags directly instead of using competitive ABPP to identify off-targets of drug candidates.

In 2020, Tate and co-workers re-evaluated the mechanism of action of α,β-unsaturated carbonyl compounds as DUB inhibitors [95], particularly of VLX1570 (Figure 7E), a compound that was put on hold in clinical trials after dose-limiting toxicity. Chemical proteomics experiments with an alkyne-tagged derivative of VLX1570 revealed CIAPIN1 as a main protein off-target. In addition, by performing various biochemical experiments in cell lysates and on whole cells, the authors identified that VLX1570 induces high-molecular-weight complexes by crosslinking various proteins through its two Michael acceptors, leading to protein aggregation, which likely contributes to its toxicity.

**Figure 8:**
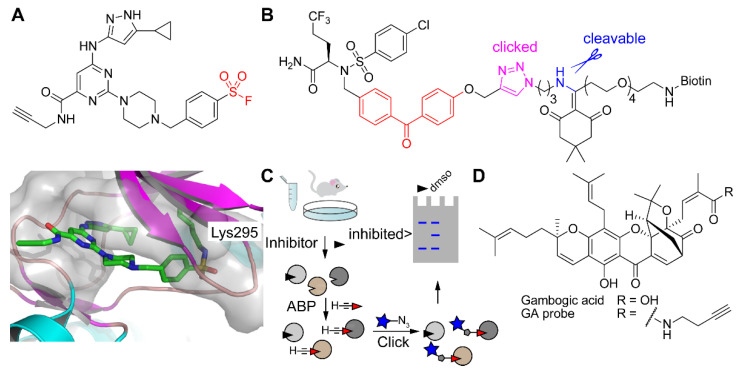
Detection of ABP binding sites and competitive ABPP. (**A**) Structure of a sulfonyl fluoride probe for kinases (upper; reactive group in red) and a co-crystal structure with Src kinase (lower panel) in which Lys295 has reacted with the sulfonyl fluoride. (**B**) Avagacestat-derived probe with a photoreactive group (in red), pre-clicked onto a biotin-azide reagent with an incorporated hydrazine-sensitive cleavable linker (cleavable bond in blue). (**C**) Schematic representation of competitive ABPP with click chemistry. Treatment with an inhibitor will block the active site on one or multiple targets, after which a broad-spectrum ABP is added to measure residually active enzymes. (**D**) Structure of gambogic acid (GA) and an alkyne-conjugated GA probe.

For mechanism-based ABPs, it is generally clear which amino acid side chain engages in a chemical reaction with the reactive electrophile (see, for example, Figure 1B,C). However, some reactive groups are more promiscuous. These include sulfonyl fluorides, which can react with various nucleophilic amino acid side chains [96], and photoreactive groups, such as diazirines and benzophenones, which undergo CH or heteroatom-H insertion when activated [8]. It is highly challenging to identify probe-modified sites in proteomics experiments. For example, Taunton and co-workers developed a sulfonyl fluoride-containing broad-spectrum kinase probe (Figure 8A) for profiling in live cells. Although various target kinases were identified following an enrichment and identification protocol as outlined in Figure 7A, the lysine binding site was only identified by incubating the purified model kinase Src with the chemical probe (without ensuing click chemistry) followed by tryptic digestion and LC-MS/MS analysis. This binding site was further confirmed by crystallography (Figure 8A).

An interesting example of binding site identification was reported by Gertsik et al., who used a photoreactive derivative of the γ-secretase inhibitor avagacestat, pre-clicked onto a biotin-azide with a hydrazine-labile cleavable linker (Figure 8B) [97]. Reaction of this probe with membrane preparations containing the γ-secretase protein complex was followed by enrichment, elution from streptavidin beads using the cleavable linker, tryptic digestion, and LC-MS/MS analysis. Note that the cleavage of the linker not only makes the modification smaller but also results in an extra positive charge on the modified peptide, which likely helps ionization and thereby enhances MS signal intensity. The probe insertion site was mapped to Leu282 in the presenilin catalytic subunit of γ-secretase. Importantly, this information was then used in combination with molecular dynamics simulations to provide a model of avagacestat binding in an allosteric pocket.

### 4.4. Inhibitor Discovery by Competitive ABPP

Within the ABPP field, competitive ABPP has become an especially powerful application to evaluate enzyme inhibitors within a complex proteome, such as a cell lysate, a live cell, or an animal model (Figure 8C) [98]. In this manner, an inhibitor can be evaluated against multiple enzymes simultaneously, providing information about its selectivity.

Sieber and co-workers discovered β-lactones as covalent inhibitors of ClpP, a protease that regulates virulence in *Staphylococcus aureus* [99]. In a follow-up work, aimed at obtaining improved inhibitors, β-lactones with various structures were synthesized and evaluated by competitive ABPP using live *S. aureus* cells and an alkyne β-lactone probe to detect residually active ClpP in live cells [100]. Detection was performed after cell lysis, click chemistry, and gel-based fluorescent scanning.

Adibekian and co-workers discovered that the xanthone natural product gambogic acid (GA; Figure 8D) lowers levels of sphingosine-1-phosphate (S1P) in MCF-7 breast cancer cells [101]. S1P suppresses ceramide-induced apoptosis and lowering its cellular levels would be attractive for cancer treatment. The authors found that GA functions by targeting a cysteine in the small subunit B of serine palmitoyl transferase complex, which performs the first step in the biosynthesis of S1P: the transfer of a palmitoyl to serine. However, GA is rather promiscuous and binds to cysteines of many proteins. A GA-based chemical probe (Figure 8D) was used in competitive ABPP with other structurally related xanthones. Interestingly, the main hit displayed much less promiscuous reactivity, while still leading to a decrease in cellular S1P levels.

### 4.5. In Situ Fluorescence Microscopy

**Figure 9:**
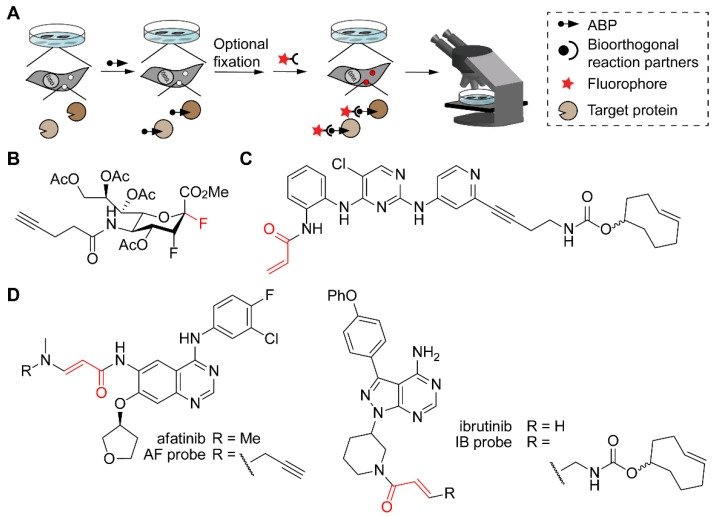
ABPP with fluorescence microscopy as read-out, which provides information on the (subcellular) location of the ABP targets. (**A**) Schematic representation of the workflow. Cells in culture are treated with an ABP. Depending on the bioorthogonal reaction, fixation may be necessary (e.g., in case of toxic conditions or non-cell permeable reagents). Bioorthogonal reaction then allows the introduction of a fluorophore, after which cells are imaged by a fluorescence microscope. (**B**) Structure of a fluorosialyl fluoride probe (reactive site in red). Note that the ester groups will be hydrolyzed by intracellular esterases to convert this molecule into the active probe. (**C**) Structure of the ERK1/2 targeting probe designed by Heightman et al. (reactive group in red) with a TCO as the bioorthogonal handle. (**D**) EGFR-targeting probe based on the drug afatinib (left) and BTK-targeting probe based on ibrutinib (right).

In all above applications, cells or tissues are eventually lysed before analysis, thereby losing spatial information. However, various studies have also used bioorthogonal reactions in ABPP with fluorescent microscopy as read-out in order to localize the target enzymes within the cell. Generally, the workflow consists of treating cells in culture with a cell-permeable ABP containing a bioorthogonal handle. After washing free probe away, cells may be fixed first or live cells may be directly subjected to a bioorthogonal reaction with a tag that enables fluorescence detection (Figure 9A).

In 2013, Wong and co-workers reported the design, synthesis, and application of a cell-permeable ABP for sialidases [102]. It consisted of an alkyne-linked 3-fluorosialyl fluoride that was peracetylated and esterified in order to render it cell permeable (Figure 9B). After treatment with this probe, cells were fixed, subjected to CuAAC with a biotin-azide reagent, and then treated with FITC-streptavidin. This procedure was not only effective in visualizing the various subcellular localizations of human neuraminidases overexpressed in HEK293T cells but also in detecting neuraminidase at the surface of viral particles in influenza virus-infected cells.

Many cells express endogenously biotinylated proteins. Therefore, detection with a fluorophore-conjugated reaction partner is preferred over the use of biotin. Heightman and co-workers decorated a covalent inhibitor of the extracellular signal-regulated kinases 1 and 2 (ERK1/2) with a TCO as the bioorthogonal handle (Figure 9C) [103]. After exposing live SW620 cells, a colon cancer cell line with an activated ERK1/2 pathway, first to probe and then to tetrazine-fluorophore, cells were fixed, washed and subjected to fluorescence microscopy. Importantly, the fast reaction kinetics with the tetrazine-conjugated fluorophore allowed the use of only a 3-fold excess of tetrazine over TCO-tagged probe, minimizing the background from free fluorophore, and a clear signal in probe-treated cells was observed. However, the signal could not be competed away with a covalent ERK1/2 inhibitor, because of off-target labeling by the ABP, as confirmed by SDS-PAGE analysis. This underlines the importance of highly selective ABPs for this application.

In a similar approach, Guo et al. designed ABPs based on the clinically approved kinase drugs afatinib and ibrutinib (Figure 9D) [104]. For the alkyne-conjugated probe, cells were fixed before performing CuAAC, but for azide- and TCO-conjugated probes, bioorthogonal reaction with cyclooctyne or tetrazine derivatives, respectively, took place on live cells. Fluorescence microscopy revealed cell membrane staining for the afatinib probe, which targets the epidermal growth factor receptor (EGFR), and cytosolic staining for the ibrutinib probe, which targets Bruton’s tyrosine kinase (BTK). Notably, pretreatment with specific inhibitors resulted in disappearance of the signals in the microscopy images, confirming on-target probe reaction.

## 5. Conclusions and Future Perspectives

The use of bioorthogonal chemistry has proven advantageous in ABPP for various reasons. Introducing molecular tags for experimental read-out directly onto an ABP may result in large molecules with unfavorable physicochemical properties. For in cell applications, for example, these labels may diminish cell uptake, introduce steric clashes with the protein of interest, or cause mislocalization of the ABP due to the intrinsic subcellular accumulation properties of the tag itself. The use of mini-tags followed by a late-stage labeling approach using bioorthogonal chemistry can circumvent these issues. An additional experimental benefit of the two-step labeling approach is the possibility to flexibly introduce tags onto biomolecules from one single experimental sample. To this end, an ABP-treated biological sample is divided into several aliquots and one part is labeled in a second step with, for example, a fluorophore for visualization, whereas the other part is conjugated to an affinity tag for isolation of the target proteins. As the labeled proteins arise from the same sample and the same bioorthogonal ligation reaction with the only difference being the reporter group, any experimental variation in labeling caused by the nature of the tag is minimized.

Although various bioorthogonal reactions have been implemented in ABPP, CuAAC remains the most popular method for introduction of a detection tag, because of the minimal size of both reaction partners and the excellent kinetics and selectivity of the reaction. However, ABPP may benefit from the wide variety of other bioorthogonal reactions that have been developed to date, for example those that exhibit faster kinetics, which would lead to lower reagent consumption and possibly lower background signals. Another interesting development in this regard are so-called turn-on fluorophores. These are quenched tags that only become fluorescent after the bioorthogonal reaction has proceeded. Examples include fluorophore-conjugated tetrazines [71,105,106], azido-coumarines [107], and azido-BODIPY dyes [108]. Such probes may be beneficial for imaging ABP targets, because background fluorescence in these cases is minimal. Depending on the type of conjugation, these reactions may need to take place on fixed cells, or they can be performed in live cells or in vivo.

Performing bioorthogonal reactions in vivo remains complex, because it places extra restrictions on the stability and reactivity of the reactants. For this reason, ultrafast bioorthogonal reactions, such as the tetrazine cycloaddition with TCO, are needed to allow conjugation in vivo even at extremely low reagent concentrations. Thus far, ABPP in combination with bioorthogonal chemistry has—to our knowledge—not yet been applied in vivo, but various examples exist for so-called ‘pretargeted radioimmunotherapy’ [109,110]. In classical radioimmunotherapy, the radioactively labeled antibody displays unfavorable pharmacokinetics, resulting in slow clearance from the bloodstream and higher radiation exposure to unwanted sites. In pretargeted radioimmunotherapy, the antibody is coupled to, for example, TCO. The radioisotope, coupled to a small-molecule tetrazine, is then administered later to ensure faster clearance from the body. We expect that similar options will be possible for ABPP-mediated PET imaging, facilitated by these fast cycloaddition reactions.

Combinations of bioorthogonal reactions, which allow parallel use of ABPs for different targets, have only sparsely been used (see Section 3). However, the multitude of available bioorthogonal chemistries now allows combinations that are compatible with each other. For more information on compatible bioorthogonal reactions, we refer to two recent review articles that have appeared elsewhere [23,83].

Besides ligation of reporter tags, bioorthogonal chemistry now also enables the de-conjugation of specific cargo molecules. Termed ‘click-to-release’, these approaches rely on specific chemistry that leads to cleavage of a bond concomitant with the bioorthogonal reaction. They have been implemented for the release of prodrugs or drugs from antibody-drug conjugates [28,111]. Such click-to-release chemistry has recently also been applied in humans in a first phase 1 clinical trial (https://clinicaltrials.gov/ct2/show/NCT04106492). The therapy is designed to reduce the systemic toxicity of the anticancer drug doxorubicin. To this end, a solid tumor is injected with a tetrazine-conjugated polymer and the patient is treated with a TCO-caged doxorubicin analog that displays much lower cytotoxicity, and is only locally released when it encounters the polymer inside the tumor. We think that such click-to-release approaches may also find future application in ABPP, for example, to image disease-related enzymes while simultaneously releasing disease-modulating drugs.

Despite the advances in bioorthogonal chemistry, new reactions are still desired in order to increase kinetics, enable compatibility, and facilitate novel applications, such as click-to-release. We believe the field of ABPP will grow in concert and will greatly benefit from the developments in bioorthogonal chemistry that may arise in the future.
